# Evaluation of Adaptive Feedback in a Smartphone-Based Serious Game on Health Care Providers’ Knowledge Gain in Neonatal Emergency Care: Protocol for a Randomized Controlled Trial

**DOI:** 10.2196/13034

**Published:** 2019-07-26

**Authors:** Timothy Tuti, Niall Winters, Naomi Muinga, Conrad Wanyama, Mike English, Chris Paton

**Affiliations:** 1 Department of Education University of Oxford Oxford United Kingdom; 2 Kellogg College University of Oxford Oxford United Kingdom; 3 KEMRI-Wellcome Trust Research Programme Nairobi Kenya; 4 Nuffield Department of Medicine University of Oxford Oxford United Kingdom

**Keywords:** neonatal mortality, education, emergency medical services, global health, smartphone, feedback, health workforce, developing countries

## Abstract

**Background:**

Although smartphone-based clinical training to support emergency care training is more affordable than traditional avenues of training, it is still in its infancy and remains poorly implemented. In addition, its current implementations tend to be invariant to the evolving learning needs of the intended users. In resource-limited settings, the use of such platforms coupled with serious-gaming approaches remain largely unexplored and underdeveloped, even though they offer promise in terms of addressing the health workforce skill imbalance and lack of training opportunities associated with the high neonatal mortality rates in these settings.

**Objective:**

This randomized controlled study aims to assess the effectiveness of offering adaptive versus standard feedback through a smartphone-based serious game on health care providers’ knowledge gain on the management of a neonatal medical emergency.

**Methods:**

The study is aimed at health care workers (physicians, nurses, and clinical officers) who provide bedside neonatal care in low-income settings. We will use data captured through an Android smartphone-based serious-game app that will be downloaded to personal phones belonging to the study participants. The intervention will be adaptive feedback provided within the app. The data captured will include the level of feedback provided to participants as they learn to use the mobile app, and performance data from attempts made during the assessment questions on interactive tasks participants perform as they progress through the app on emergency neonatal care delivery. The primary endpoint will be the first two complete rounds of learning within the app, from which the individuals’ “learning gains” and Morris G intervention effect size will be computed. To minimize bias, participants will be assigned to an experimental or a control group by a within-app random generator, and this process will be concealed to both the study participants and the investigators until the primary endpoint is reached.

**Results:**

This project was funded in November 2016. It has been approved by the Central University Research Ethics Committee of the University of Oxford and the Scientific and Ethics Review Unit of the Kenya Medical Research Institute. Recruitment and data collection began from February 2019 and will continue up to July 31, 2019. As of July 18, 2019, we enrolled 541 participants, of whom 238 reached the primary endpoint, with a further 19 qualitative interviews conducted to support evaluation. Full analysis will be conducted once we reach the end of the study recruitment period.

**Conclusions:**

This study will be used to explore the effectiveness of adaptive feedback in a smartphone-based serious game on health care providers in a low-income setting. This aspect of medical education is a largely unexplored topic in this context. In this randomized experiment, the risk of performance bias across arms is moderate, given that the active ingredient of the intervention (ie, knowledge) is a latent trait that is difficult to comprehensively control for in a real-world setting. However, the influence of any resulting bias that has the ability to alter the results will be assessed using alternative methods such as qualitative interviews.

**Trial Registration:**

Pan African Clinical Trials Registry PACTR201901783811130; https://pactr.samrc.ac.za/TrialDisplay. aspx?TrialID=5836

**International Registered Report Identifier (IRRID):**

PRR1-10.2196/13034

## Introduction

### Background

In low-income contexts such as Sub-Saharan Africa, the need for health workers to provide care for themselves is more frequent than that in middle- and high-income settings and can be associated with negative neonatal outcomes [1]. Of the estimated 2.9 million neonatal lives (from birth through day 28) lost each year globally, Sub-Saharan Africa has the highest overall risk of death within the first 24 hours of life and accounts for 37% of the global neonatal deaths [1]. This is compounded by severe workforce shortage, coupled with the health workforce skill imbalance and maldistribution, and a lack of training opportunities [2,3]. Additional training is needed to better prepare health providers in these facilities to provide effective emergency pediatric and neonatal care [4,5]; however, unfortunately, face-to-face training costs between US $80 to $300 per person per day and is difficult to deliver at scale [6]. A face-to-face course developed in Kenya (Emergency Triage, Assessment and Treatment plus admission care [ETAT+]) [7,8] has been successfully used to provide training in Kenya as well as eight other low-income countries [9]. ETAT+ has already been used to train over 5000 health care workers and 2000 medical students across Kenya, Uganda, Rwanda, Zimbabwe, Zambia, Malawi, Tanzania, Sierra Leone, and Myanmar. It is part of the undergraduate medical training curriculum in medical schools in East Africa [10]. The aim of the ETAT+ is to familiarize health workers with clinical guidelines and the necessary knowledge and skills for triaging all sick children when they arrive at a health facility into emergency cases with priority signs or nonurgent cases and to provide emergency treatment for those with life-threatening conditions [11]. Only a small fraction of trained health providers have received the basic requisite skills training for newborn resuscitation in this context [12,13]. New strategies are therefore required to improve access for over 1 million health providers across Africa, and training approaches need to be updated efficiently as guidelines change and be able to capture data on how many health workers are trained [14,15].

There is little evidence to support the implementation of learning interventions that are relevant to the context of low-income settings, which takes into account health workers’ initial and continuing clinical training needs and adapts learning content in light of skill mastery and performance as they continue to develop knowledge through it [15-18]. Within low-resource contexts, investigation of learner models (ie, a cognitive model that tries to model observed student learning behaviors) needed to support tailored instruction based on predicted learner’s skill mastery in clinical settings is required [19], as adaptive instructional support has been shown to significantly outperform teacher-led large-group instruction, nonadaptive computer-based instruction, and paper-based instruction in producing learning gains in high-resource settings outside clinical care [19].

Several health care training apps have been developed to date, and the approaches used therein can broadly be divided into two categories. Some replicate existing teaching strategies “on a screen,” for example, by providing questions and answers for exam practice or displaying of textbook graphics. Others take advantage of features specific to mobile devices, examples of which include the ability to respond to user choices with different pathways or the use of animations with which the user can interact [15]. Serious games, which are digital games with a specific, applied purpose (other than entertainment) that can be played on mobile phones, are one such way of providing training with the potential to affect health outcomes [20]. The rationale for using serious games is that, similar to “first-person” computer games, emergency care training should enable health workers to follow highly structured pathways (such as clinical care algorithms) with pieces of information (cues) sought at each step, which determine the correct actions to perform. With both clinical training and performance in computer games, executing cue-response sequences perfectly, rapidly, and automatically demonstrates mastery. This type of mastery has been shown to support effective clinical care delivery, but the required frequency of rehearsal in this approach is difficult and expensive to maintain for face-to-face training [21]. By using a serious gaming approach, users may be more motivated to repeatedly play the serious game, using incentives such as rewards, increasing difficulty, and scores—techniques that have been successfully used to encourage repeated gameplay in nonserious computer games. There is a scarcity of evidence from studies evaluating and assessing serious gaming approaches using smartphones in health care training in low-income settings, but this topic was recently highlighted in the most recent systematic review [22,23].

Efforts to implement innovative educational interventions, which increase the number of well-trained health providers, are yet to capitalize on digital learning approaches that model learners’ knowledge states to provide individualized instruction and have been shown to have considerable positive effects on learning outcomes in other subject areas [5,24,25]. The Life-Saving Instruction for Emergencies (LIFE) project is developing an approach to create serious games for low-cost smartphones initially to provide training for care of very sick newborns and children. The game evolves the scenario-based teaching model that is used in the traditional face-to-face ETAT+ course already described.

The potential for utilizing the digital and reusable nature of interventions such as LIFE, to personalize the way health workers learn and receive feedback on their performance on which they can base future learning, achieve learning objectives, and develop their skills is still underexplored and unknown [18,22]. Additionally, the use of smartphones as experimental tools offers access to a wider pool of study participants due to their ubiquitous nature [26]; can minimize the cost of implementing, evaluating, and scaling an educational intervention such as LIFE in a resource-constrained context [15], and has been shown to raise learners’ interest in knowledge interventions [27].

### Objectives

The primary objective of this randomized experiment is to investigate whether adaptive individualized feedback is superior to standardized feedback in smartphone-based emergency neonatal care training. We hypothesize that health care providers randomized to receive adaptive feedback will have a significant improvement in learning gains as compared to those randomized to receive standardized feedback.

## Methods

### Study Design

The study (trial registration: Pan African Clinical Trials Registry PACTR201901783811130) will have a parallel-group double-blinded randomized experiment design with an allocation ratio of 1:1. The participants will be randomized to the intervention or control group when they launch the android-based training app for the first time on their individual smartphone devices.

### Eligibility Criteria

Health care workers who are in nursing, clinical, or medical professional cadres that offer bedside patient care are included. Additionally, health care workers in practice and those in training are eligible for inclusion into the experiment. Health care workers who have retired from clinical practice and participants who are not health care workers will be excluded from the study.

### Study Setting and Recruitment

This study does not have a specific physical study site; it focuses on low-income countries that stand to benefit from ETAT+ training. Distribution of the intervention is through the Google Play Store, with initial efforts directed toward physical recruitment in Kenya. Recruitment of study participants will be through three main avenues: (1) remotely, by raising awareness of the Android Google app on social media platforms and online health networks forums every couple of weeks during the study duration to promote voluntary, self-enrolment for use of the training tool; (2) use of snowballing sampling by ETAT+ trainers, previous study participants, and lecturers in medical sciences at local Kenyan universities to suggest that (and encourage) other health workers to self-recruit into the study; and (3) use of a study sample from the purposive selection strategy that will be actively convened once every month, with a unique set of participants to ensure the participant recruitment targets are diverse and met. Snowballing sampling is a type of convenience sampling where a group of people (in our case, already recruited participants) recommend potential participants for the study [28]. The cycle of recommendation of participants for inclusion into the study continues during the study duration. This also serves as an indicator of LIFE intervention adoption. The first avenue includes platforms that are focused on health capacity building in the global south in line with LIFE’s focus on low-income contexts. Avenues 2 and 3 will take place at the following sites: Kenyatta National Hospital - Neonatal Nurse Training Unit; College of Health Sciences, University of Nairobi; Kenya Medical Training College (Nairobi); and Gertrude’s Garden Children’s Hospital Nurse Training School (Nairobi).

### Intervention

The intervention in this study involves adaptive, personalized, and immediate feedback that is provided while learning through a smartphone-based serious gaming Android app. The content to be learned is based on ETAT+ guidelines, a course that is already offered in nine low-income countries [7,8,10]. The intervention will be available on the Google Play Store where it will be publicly accessible, downloadable, and installable to any compatible android-based mobile device. All study participants will receive a link to the mobile app hosted on the Android Play Store. The LIFE app was designed for Android’s Target SDK 19 as the minimum version of Android supported as of February 2019 (which targets 100% of Android devices) [29]. Given that the intervention has undergone alpha and beta testing on health care providers’ smartphones since October 2017 in Kenya, we are confident of the stability of the app.

The personalized immediate feedback to be given to the experimental group participants is designed to arouse meaningful immersive learning experiences from continuous interaction between the learners and the smartphone-based training [30,31]. This adaptive feedback will be provided to experiment group participants after each incorrect attempt at a learning task with three cascading detail levels based on predicted probability that the learner’s next attempt is going to be correct. The wording of the feedback provided is dependent on the number of incorrect choices the learner had selected and the actual incorrect choices themselves. The control group study participants will receive standardized nonpersonalized immediate feedback after each incorrect attempt at a learning task, with the feedback on the first incorrect attempt asking the learner to retry and the feedback on the second attempt providing a detailed explanation of the correct choices to select.

The LIFE app is a measurement tool; at the end of successful completion of a learning session, the platform provides performance scores based on whether each learner’s response to the learning task was correct on the first attempt.

This methodology mimics a common approach in experimental design known as A/B testing, which is used to optimize concepts such as feedback or user interface on digital platforms [32,33]. In particular, the experiment described here emulates A/B tests’ commonly used Hypothesis Experiment Data‐Driven Development model, whose description and use are detailed elsewhere [34]. Such an experimentation approach can be used for digital health interventions where there is need for data-driven decision making on inclusion of features and the data are trustworthy, but assessment of the added value of features on the digital platform is difficult [33].

### Outcomes

The primary endpoint for both arms of the experiment will be the presence of two complete rounds of learning sessions using LIFE, the former is the pretest round and the latter is the posttest round. Both scores will be converted into percentages. From the pre-post scores, the study’s main outcome—the learning effect size (*d*), [35]—will be calculated as follows:

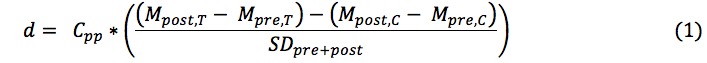



where the pooled SD is defined as:

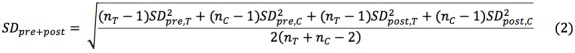



The bias adjustment is provided by the formula:

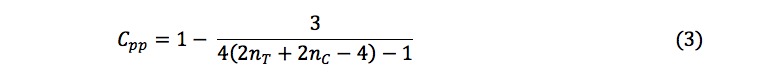



where *n*_*T*_ is the sample size of the treatment group, *M*_*pre,T*_ is the pretest mean for the treatment group, *M*_*post,T*_ is the posttest mean for the treatment group, *SD*_*pre,T*_ is the SD of means for pretest in the treatment group, *SD*_*post,T*_ is the SD of means for posttest in the treatment group, *n*_*C*_ is the sample size of the control group, *M*_*pre,C*_ is the pretest mean for the control group, *M*_*post,C*_ is the posttest mean for the control group, *SD*_*pre,C*_ is the SD of means for pretest in the control group, and *SD*_*post,C*_ is the SD of means for posttest in the control group.

The effect size from equation from (1) is referred to as *Morris G* [35] and represents the mean difference between the study groups. Because of randomization, this calculation allows to control for pre-existing differences among learners (eg, intelligence quotient level), to estimate treatment effectiveness even when the treatment and control groups are not equivalent, and to consider the variances of both pretest and posttest scores. This contrasts with other forms of effect-size calculation such as Hedges G and Becker D, which only use pretest or pooled variances [35]. In this model, the pretest and posttest variances are assumed to be homogeneous.

Secondary outcomes that will be assessed are time difference between each round of learning sessions, the number of times a learner has encountered a learning task up to the current opportunity (ie, cumulative attempts on learning task per learner), the time spent on each learning task, and the level of feedback provided. These calculations will be performed in Python, version 3.6.8 (Python Software Foundation, Wilmington, DE).

### Participant Timeline

Enrolment of study participants began on February 1, 2019, and will continue up to July 31, 2019. Because the rollout of LIFE’s intervention in this study is based on the principles and outcomes of an implementation study [36] and informed by self-regulated self-directed learning [37-39], it seeks to understand and work within real-world conditions, rather than trying to control for adoption, acceptability, coverage, and sustainability conditions or remove their influence on the study outcome [36]. Subsequently, to optimally maintain fidelity to LIFE’s planned scale-up, no training sessions are planned for the study participants. Although LIFE is designed for low-income contexts, we set no limit by geographical coverage for self-directed health care providers who might be interested in undertaking this training. However, for primary outcome analysis, participants from high-income countries will be omitted. Participants without any geographic location data (due to refusal to grant the LIFE app the required Android permissions) will be assumed to be from developing countries, given that our recruitment efforts are directed toward professional groups in these countries.

### Sample Size Calculation

Similar interventions in other subject domains have been found to have an mean effect size of 0.22 (95% CI 0.16-0.27) from a meta-analytic fixed-effects model [24]. To detect an effect size of 0.22 with a two-sided 5% significance level and a power of 80%, a sample size of 83 participants per group, who reach the primary endpoint of the study, is necessary. A sample size calculation for a one-way ANOVA, together with one-sample and paired-sample *t* test analysis using the same effect, power, and significance parameters produce the same required sample size. We anticipate recruiting this number of participants (N=166) in 6 months. The sample size calculation formula is given in the Multimedia Appendix 1.

Based on the alpha and beta tests of the LIFE version, we are assuming a 50% dropout rate of study participants, with drop-out defined as the incomplete or single use of the LIFE smartphone app. We plan to recruit at least 332 participants to account for this dropout rate. To encourage repeated usage of LIFE, participants will receive up to three email reminders from the time they are enrolled into the study, spread over 3 weeks. Demographic data will be collected in the app during its initial use by study participants at the end of the first learning session. Thus, for learners who drop out before completing the first session, or chose not to fill in those data, no demographic data will be available. Logistic regression analyses will be performed on these data to evaluate whether there is a systematic bias caused by attrition of study participants that affects the interaction between the study groups and performance, exposure to previous ETAT+ training, clinical cadre, age of participant, and level of experience. In an effort to ensure participant recruitment is at par with the required study sample size, a monthly target of 30 participants, evaluated at the end of each month, has been set for this study.

### Randomization

For allocation of the participants, an in-app algorithm will randomly generate a value of zero or one when the Android-based smartphone app is launched for the first time. This will determine whether the participant is allocated to the control or experiment group. It will also blind both the study participants and intervention staff to the group allocation of participants during the experiment, but not at the analysis stage. Sequence generation for random allocation is a computerized procedure pegged on a single instance (ie, smartphone app installation) that mimics a coin-tossing procedure. Therefore, use of permuted blocks of random sizes to assign participants to either the control or experimental group is not possible and will not be implemented.

### Statistical Methods and Planned Analysis

For the primary endpoint, we will use the Morris G effect size to analyze the differences among group means in the study population. This will be assessed after the performance from the second round of training through the mobile app has been recorded. Secondary analysis will be conducted using regression analysis, with learning gains as the dependent variable and the aforementioned secondary outcomes as the independent variables, to evaluate their effect on learning gains. Learning gains, defined as the amount the health care providers learned divided by the amount they could have learned [40], will be calculated with the formula given in Equation 8 in Multimedia Appendix 1.

Due to the definition of the primary learning outcome used in this study, the outcome cannot be computed for study participants whose dropout is characterized by the lack of at least two complete learning sessions. Without a postbaseline assessment, “intention-to-treat” analysis cannot be performed for dropout cases unless we impute outcomes, which tends to produce biased estimates [41]. This study will not be able to conduct an intention-to-treat analysis. However, dropout numbers will be reported in relation to those who reached the study’s primary endpoint, and their implications will be discussed with regard to self-regulated self-directed learning [37-39].

Qualitative interviews will be conducted in parallel with the experiment for participants who have reached the primary endpoint and those who drop out. These interviews will be used to explore how self-regulation in learning affected the use of the smartphone-based learning platform and to provide context for interpreting the observed learning outcomes from this study apart from offering evidence for tool validation.

### Data Management

The primary data collected from the study participants’ Android smartphone app will be held on their devices with a backup copy synchronized to *Google Firebase*, a secure distributed online database server, after transmission in an encrypted format. During data collection, transcripts and recordings of interviews after the experiment will be stored on encrypted password-protected USB devices and transferred to secure password-protected servers in Kenya and Oxford. De-identified data will be shared with the Kenya Medical Research Institute (KEMRI)-Wellcome Trust Research Program and University of Oxford for agreed analyses, with only named investigators having access to the data.

### Ethics and Dissemination

The analyses described in this protocol have been approved by the KEMRI’s Scientific and Ethical Review Committee (#3444) and the Central University Research and Ethics Committee of Oxford University (#ED-CIA-18-106). Individual patient consent will be elicited from within the mobile app before collection of any demographic data in addition to using explicit Android permission requests. This approach of obtaining in-app informed consent is not uncommon in medical research; it has been described in detail in a previous systematic review [42] as well as specifically in mobile app–based research [43]. The results of this analysis will be shared with the Kenyan Ministry of Health, submitted to peer-review publications, and presented at international conferences.

## Results

This project was funded in November 2016. Data were collected from February 2019 up to July 2019. As of July 2019, we enrolled 541 participants, of whom 238 reached the primary endpoint, with a further 19 qualitative interviews conducted to support evaluation (New-Born Unit in-charge nurses, n=4; Paediatric Intensive Care Unit nurses, n=5, final-year medical students already doing clinical rotations, n=4; and student clinical officers and nurses already doing clinical rotations, n=6). Full analysis will be conducted once the number of required participants is met, and the results are expected to be published by Spring of 2020.

Based on the education literature [24,44,45], an effect size of approximately 0.2, 0.5, and 0.8 will be considered small, moderate, and large, respectively. These thresholds represent the magnitude of effect and reflect our assumption that a statistically significant result is not necessarily important or meaningful; for example, for an effect size of 0.2, the difference between the study groups is trivial even if it is statistically significant [44,46].

## Discussion

This study will be used to explore the effectiveness of adaptive feedback for learning in smartphone-based training for health care workers in a low-income setting. This aspect of medical education is a largely unexplored topic. In this randomized experiment, the risk of performance bias across arms is moderate, given that the active ingredient of the intervention (ie, knowledge) is a latent trait that is difficult to comprehensively control for in a real-world setting. However, the influence of any resulting bias’s ability to alter the results will be assessed within this study using qualitative interviews with study participants.

To help minimize attrition in the study, participants in all study arms who have not reached the primary endpoint will receive up to three email reminders to use the smartphone app from the time they are enrolled into the study, every couple of weeks. The email message will also include a running counter of how many other participants from across the arms of the experiment have successfully played the game so far. The locally accessible study participants will be reimbursed for the mobile data costs incurred when downloading the game if that proves to be a barrier. Whether mobile data charges are a barrier to recruitment of participants and playing the game numerous times will be evaluated through qualitative interviews on an ongoing basis throughout the study period. In case the participant recruitment does not meet the monthly targets, where possible, nonmonetary incentives (such as provision of clinical guidelines protocol booklets) will be phased in to motivate participation in the study.

## Appendix

Multimedia Appendix 1 Sample size calculation formula.

## Abbreviations

LIFE: Life-Saving Instruction for Emergencies
KEMRI: Kenya Medical Research Institute
