# Supplementary material for: Evaluation of Adaptive Feedback in a Smartphone-Based Serious Game on Health Care Providers’ Knowledge Gain in Neonatal Emergency Care: Protocol for a Randomized Controlled Trial
Source: JMIR Res Protoc. 2019 Jul 26;8(7):e13034. doi: 10.2196/13034 (PMC6688438; doi:10.2196/13034)
Supplement: Multimedia Appendix 1 [file resprot_v8i7e13034_app1.pdf]

Sample size calculation:

Suppose k groups each have a normal distribution and equal means ($\mu_1 = \mu_2 = \cdots = \mu_k$). Let $n_1 = n_2 = \cdots = n_k$ denote the number of subjects in each group and let N denote the total sample size of all groups. Let μ_W denote the weighted mean of all groups i.e.

$$\mu_W = \sum_{i=1}^{k} \left(\frac{n_i}{N}\right) \mu_i \qquad (4)$$

Where:

n_i = number of observations in group i

N = total number of observations

μ_i = the mean of group i

The ratio of the mean square between groups to the mean square within groups is a F-distribution, with two parameters matching the degrees of freedom of the numerator mean square and the denominator mean square. When the null hypothesis of mean equality is rejected, that ratio has a noncentral F-distribution which is dependent on the non-centrality parameter, λ calculated as:

$$\lambda = N \frac{\sigma_m^2}{\sigma^2} \qquad (5)$$

Where:

σ^2 = error variance within groups

σ_m^2 = error variance between groups

and

$$\sigma_m = \sum_{i=1}^{k} \frac{n_i(\mu_i - \mu_w)^2}{N} \qquad (6)$$

Power of the study is calculated as:

$$Power = 1 - F(f_\alpha; k-1, \upsilon, \lambda\,) \qquad (7)$$

Where:

ν = degrees of freedom for the error, equal to k * (n_i – 1)

k = number of levels (groups).

$F(f_\alpha; k-1, \upsilon, \lambda)$ = a continuous distribution function of F-distribution, with k-1 degrees of freedom for the numerator, ν denominator degrees of freedom, and the non-centrality parameter evaluated at f_α

Within R statistical language, to derive the required sample size, this is easily translated to:

```
library(pwr)

pwr.anova.test(k = 2, f = .22, sig.level = .05, power = .8)
```

The effect size used for the power calculation is based on education literature where similar interventions in other subject domains using the experimental study design have been found to have an mean effect size of 0.22 (95% CI: 0.16 – 0.27) from a meta-analytic fixed-effects model [24].

Learning gains defined as the amount healthcare providers learned divided by the amount they could have learned [40], will be calculated with the formula given below:

$$Learning\ gain\ (c) = \begin{cases} \dfrac{post - pre}{100 - pre} & post > pre \\ drop & post = pre = 100 = 0 \\ 0 & post = pre \\ \dfrac{post - pre}{pre} & post < pre \end{cases} \tag{8}$$

This helps to minimise the low test-score bias in calculation of individual health providers knowledge gain and makes interpretation of learning scores is straightforward by ensuring the learning gain neither falls within a non-symmetric range of scores nor includes infinity values [40]. These limitations are present in simplified calculations of gain score e.g. gain=post-score − pre-score [40].
